# A PL1 family pectate lyase *CP966_RS08110* gene was the pathogenic factor of *Streptomyces galilaeus* 5T-1 causing potato common scab

**DOI:** 10.3389/fmicb.2024.1469709

**Published:** 2024-11-27

**Authors:** Cuiwen Zhang, Chengde Yang, Mengjun Jin, Zhonghong Feng, Richard Osei, Fengfeng Cai, Ting Ma, Yidan Wang

**Affiliations:** ^1^College of Plant Protection, Gansu Agricultural University, Lanzhou, China; ^2^Plant and Bacterial Diversity Laboratory of Gansu Province, Lanzhou, China

**Keywords:** *Streptomyces galilaeus*, pectate lyase, prokaryotic expression, condition optimization, gene knockout

## Abstract

Pectate lyases (PL), as important polysaccharide lyases, play an important role in the infection of host plants by pathogenic. A previous study found that the PL gene *CP966_RS08110* was up-regulated in the interaction between *Streptomyces galilaeus* 5T-1 and potatoes. In this study, *S. galilaeus* 5T-1 was used as the study object, and its gene function was investigated using bioinformatics analysis, prokaryotic expression, and CRISPR-Cas9 technology. The previous results showed that the pectate lyase *CP966_RS08110* gene of *Streptomyces galilaeus* 5T-1 was up-regulated in the pathogenic process. In this study, the *CP966_RS08110* gene was cloned from the genomic DNA of *S. galilaeus* 5T-1. It encoded for a 415-residue protein with a complete PL-6 superfamily domain and Pec_lyase_C domain, which belongs to the PL1 family. The soluble protein encoded by *CP966_RS08110* was obtained successfully, which has high pathogenicity after inoculating healthy potatoes. The mutant strain △PL5T-1 with *CP966_RS08110* gene deletion was successfully obtained, and its colony morphology and pigment were not significantly different from that of wild strains, but its growth rate was slowed down, moreover, the hyaline circle formed by the mutant strain ΔPL5T-1 using pectin was significantly smaller than wild strain, and the deletion of this gene affected the infestation rate of *S. galilaeus* 5T-1. Our results confirm that the *CP966_RS08110* gene was the pathogenic factors and played a key role in process of infecting and causing potato common scab, which laid foundation for understanding the pathogenic mechanism of *S. galilaeus* 5T-1.

## 1 Introduction

Potato (*Solanum tuberosum*) is the fourth largest food crop and plays an important role in ensuring the stability of the human food supply (Stokstad, 2019). Potato common scab (PCS), a global issue that has adapted to different soil types and is caused by pathogenic *Streptomyces* spp. which is posing more harm every year (Nguyen et al., 2022). As of February 2021, there are 960 valid species of Streptomyces have been published, of which more than 35 have been identified as pathogenic, including *S. scabies*, *S. acidiscabies*, *S. turgidiscabies*, *S. europaeiscabiei*, *S. stelliscabiei*, *S. bottropensis*, *S. diastatochromogenes*, *S. niveiscabiei*, *S. bobili* and so forth (Lambert and Loria, 1989a,b; Miyajima et al., 1998; Bouchek-Mechiche et al., 2000; Zhou et al., 2017; Yang et al., 2017; Park et al., 2003). The most prevalent species are caused by three different Streptomyces species: *S. scabies*, which originated in the United States, *S. acidiscabies*, which causes acid scabies, and *S. turgidiscabies*, which causes convex scabies. Common symptoms of scabs include a few raised or pitted lesions on the skin of the potato tuber that resemble scabs or warts, which can affect the appearance and quality of potatoes, in addition, the process of tuber infection is accompanied by a decrease in potato resistance, which further provides the possibility for the invasion of other pathogens (Wanner, 2006; Wanner et al., 2014), resulting in a 10–30% yield reduction and significant financial losses (Li Y. et al., 2023).

The pathogenic factors of PCS mainly include toxins, enzymes and hormones. The key virulence factors of *Streptomyces* are a family of phytotoxic metabolites called thaxtomins, cyclic dipeptides (2,5-diketopiperazines) derived from the condensation of L-phenylalanine and L-4-nitrotryptophan moieties (Bignell et al., 2014). Furthermore, *Streptomyces* also secretes a variety of polymer-degrading enzymes, such as cellulase, pectinase, lipase and esterase, which cooperate to decompose plant cell walls, thus invading and spreading in host tissues. According to Fellows (1926), pathogen may emit toxins or enzymes that cause the tissue at the sick location of potato slices to turn black, and some scholars reported that esterase could also induce scab symptoms (Schottel et al., 1992). Studies on the pathogenic mechanism of *S. scabiei* have found that the single ADP-ribosyltransferase acts on guanine, resulting in the functional inactivation of target sites, which is predicted to be a virulence factor (Lyons et al., 2016). Huguet-Tapia et al. (2016) has found a gene encoding sub1 in the genome of *S. scabiei*, this gene is absent in saprophytic *Streptomyces*, but highly conserved in pathogenic *Streptomyces*, so it is speculated that the *sub1* gene is closely related to the pathogenic ability of the strain.

As the fundamental components of the plant cell wall, cellulose, and pectin serve as the first line of defense against pathogen invasion. Enzymes, as a major component of pathogenicity factors, will facilitate pathogen invasion of plants by eliminating cellulose, pectin and other plant components (de Oliveira Silva et al., 2024). To soften and break down the pectin in the host cell wall, pectin methylesterase (PME), polygalacturonase (PG), and pectate lyase (PL) cooperate (Bonnin and Pelloux, 2020), which interferes with the host plant’s first line of defense, which increases the pathogen’s affinity for the host and eventually causes tissue maceration and death (Deising et al., 2000; Heffron et al., 1995; Kikot et al., 2009). Pectate lyase (EC 4.2.2.2), also referred to as polygalacturonate lyase (PGL), is capable of cleaving the *α*-1, 4-glycosidic bonds by *β*-elimination, producing 4,5-unsaturated oligogalacturonides (Yan et al., 2024). Jiang et al. (2023) reported that deletion of the PL gene *Vd PL1-4* in *V. dahliae*, the incidence of verticillium wilt was significantly decreased. Hassan et al. (2013) reported that the mutation of *Pel N* gene of *D. dadantii* 3,937 reduced the virulence of pathogenic to chicory. Similarly, the deletion of PL gene *BcPG1 and BcPG2*also reduces the toxicity of *B. cinerea* (Have et al., 1998; Kars et al., 2005).

The majority of studies on the pathogenesis of PCS have focused on the thaxtomins (Healy et al., 2000; Lerat et al., 2009) and extracellular esterase (Raymer et al., 1990; Schottel et al., 1992), but pectinase associated with *Streptomyces* infested potato has not been reported. The PL gene *CP966_RS08110* (Sequence ID: PQ477902) was also shown to be up-regulated in the pathogenic process of *S. galilaeus* 5T-1 by earlier study (Cui, 2022). Therefore, in this investigation, *CP966_RS08110* was expressed in *E. coli* BL21 (DE3) after being cloned from the genomic DNA of *S. galilaeus* 5T-1. Based on the characteristics of the PL gene in *S. galilaeus* 5T-1, the function of the gene was investigated, offering a theoretical framework for the investigation of the pathogenic mechanism and PCS action mechanism.

## 2 Materials and methods

### 2.1 Strains, plasmids and genomes

Trelief^®^ 5α Chemically Competent Cell was purchased from Tsingke (Beijing), *E. coli* BL21 (DE3) Chemically Competent Cell was purchased from Biomed, *E. coli* ET12567 (PUZ8002) Chemically Competent Cell was purchased from Zoman, pET-28a (+) vector was purchased from Sangon Biotech, T-Vector pMD™19 (Simple) was purchased from Takara, pKC1139-tr cas9-acrIIA4-atpD (referred to as pTRIA) was gifted by Mr. Xuming Mao at Zhejiang University and the strain *S. galilaeus* 5T-1 was preserved in the Plant and Bacterial Diversity Laboratory of Gansu Agricultural University, *S. galilaeus* 5T-1 genomic DNA was extracted according to bacterial genomic DNA extraction kit. Primers were shown in Table 1.

**Table 1:** Primers were used in this study.

Primer name	Sequence 5′-3′
CP-F	ATGCTGGGTCTGCTCTGTGTC
CP-R	TCATGGGATCCTCCCTGC
08110-F	CCG**GAATTC**ATGCTGGGTCTGCTGTGCGTT (EcoR *I*)
08110-R	CCG**CTCGAG**CGGGATACGACCAGCACCAG (Xho *I*)
sense oligo	GGCGATCCGGATGATCTTCGGTTTT
antisense oligo	GGTCGCCGCTAGGCCTACTAGAAGC
Up-F	CCC**AAGCTT**CAGGTTCAAGCAGTTCTGGCACAT (Hind Ⅲ)
Up-R	GTGTTCATCGGTTCGGGGGTGTCACGGCGATATGACGGACTCT
Down-F	AGAGTCCGTCATATCGCCGTGACACCCCCGAACCGATGAACAC
Down-R	CCC**AAGCTT**CGTGGTTGTCGAGTTCGGT (Hind Ⅲ)
D-F	GGATCGCCGTGATCTGCAC
D-R	CACCACCACCACTGAGCTAG
sg-F	GAGCTAGTTGCCGATTCTGGTCT
sg-R	AGCTAGCTTCAGACGTGTCTAGTGC
aac-F	TTTATCACCACCGACTATTTGC
aac-R	TCATCTCGTTCTCCGCTCAT
Q1	CTCAACACCCTGATGAGCG
Q2	ACGATGCCCTTGTCCTGGA
F1	TGGTGAGGGAGAAGGTGAAGT
R1	CTCCAGGACAAGGGCATCGT
F2	GCGATATGACGGACTCTCACC
R2	GCCAGCAGGTTCAAGCAGTT
F3	CTGTCGATCTTTGTGTGCAG
R3	GACAGGTGCACCTCTACAAC

### 2.2 Methods

#### 2.2.1 Bioinformatics analysis

The biological characteristics of the 08110 protein were analyzed as follow. First of all, the nucleotide sequence was translated into the amino acid by ExPASy-Translate.^1^ Then, the basic physical and chemical properties were predicted using ProtParam^2^; The hydrophilicity was predicted using ProtScale^3^; The signal peptide was predicted using SignalP 4.1,^4^ transmembrane structure was predicted using TMHMM-2.0^5^; and the conservative structure domain was predicted using CDD^6^ and dbCAN^7^; secondary structures were predicted using SOPMA^8^; and the tertiary structure was predicted using SWISS-MODEL.^9^ Finally, the phylogenetic tree was constructed using MEGA-7.

#### 2.2.2 Gene cloning

Based on the previous analysis of transcriptome data of *S. galilaeus* 5T-1, the up-regulated expressed gene *CP966_RS08110* was amplified using CP-F and CP-R primers. The PCR products were detected and purified by electrophoresis on 2.0% agarose gel. The purified target fragment with A tail was cloned into pMD-19 T vector by T-A, transformed into Trelief^®^ 5α Chemically Competent Cell, and screened for positive clones on ampicillin-resistant plates. Positive clones were sent to Tsingke (China, Shanxi) for sequencing.

#### 2.2.3 Recombinant protein expression and pathogenicity determination

##### 2.2.3.1 Codon optimization

To enhance the PL gene of *S. galilaeus* 5T-1 compatibility with the *E. coli* expression system, the database *E. coli* Codon Usage Analyzer 2.1 was used to analyze the usage frequency of codon, and the *CP966_RS08110* gene was optimized by codon optimization software JCat. According to the codon bias of *E. coli* (strain K12), the codon was optimized by changing the GC content and CAI (codon adaptation index) of the original gene sequence by the substitution of the synonymous codon.

##### 2.2.3.2 The prokaryotic expression vector was constructed

The optimized *New-08110* gene was synthesized by Tsingke (Beijing), which was constructed on a pUC57 vector to form a recombinant plasmid. Primers 08110-F/08110-R were designed based on the multiple cloning sites of the pET-28a (+) vector and characterization of the target sequence, selecting the restriction enzyme sites EcoR *I* (GAATTC) and Xho *I* (CTCGAG) as insertion sites for the target fragments, and the stop codon was removed after considering the purification of the target protein. After the target fragment was amplified and purified, the target fragment and pET-28a (+) were cut with EcoR *I* and Xho *I*, then separated by 2% agarose gel electrophoresis and linked with T4 ligase. The recombinant plasmid pET-28a-*New*-*08110* was transformed into *E. coli* DH 5α. Positive clones were screened by LB (50 μg/mL kana) and verified by colony PCR and sequencing.

##### 2.2.3.3 Induced expression of protein

The recombinant plasmid pET-28a-*New*-*08110* was transformed into *E. coli* BL21 (DE3) to obtain the *E. coli* BL21 (pET-28a-*New*-*08110*) expression strain, and the plasmid pET-28a (+) was transformed into *E. coli* BL21 (DE3) to obtain the *E. coli* BL21 (pET-28a) as no-loaded strain. A single colony that was correctly identified by PCR was placed in 20 mL LB (50 μg/mL kana) at 37°C, 200 rpm, and cultured for 16–20 h to prepare the overnight cultures, which was inoculated into 50 mL LB (50 μg/mL kana) at 1% (v/v) inoculum and until an optical density at 600 nm (OD_600_) of 0.6–0.8 (logarithmic growth phase). Then, expression was induced by adding 0.8 mM IPTG and cultured at 37°C for 8 h, the non-induced strains and no-loaded strain were used as controls. After induction, the bacteria were collected and re-suspended in the lysate buffer (containing 1 mM PMSF), and then collect the sediment by centrifugation, which was detected by 12% SDS-PAGE gel and stained with Fast blue protein staining solution.

##### 2.2.3.4 Purification of His-tag protein

The target protein was purified by affinity chromatography using a Ni-NTA since the expressed protein has His-tag and specifically binds Ni^2+^ metal chelate. The protein was expressed under the optimal induced conditions, and the supernatant of lysate was collected and mixed with the sample buffer in equal volume, boiled for 10 min, and centrifuged, 0.02 mL of the supernatant was taken for electrophoresis detection.

##### 2.2.3.5 Pathogenicity of purified protein

###### 2.2.3.5.1 Potato tubers method

Potato tubers were tested for pathogenicity based on the method of Loria and Kempter (1986) with some modifications. Select healthy potato tubers, disinfect them in 75% alcohol for 1 min, then rinse 3 times with sterile water, and sterile filter paper to absorb excess water, and gently scratch with a sterile needle to cause micro-wounds. A 5 mm sterile filter paper soaked with purified protein was placed over the wound. The potatoes were placed at 28°C and relative humidity was greater than 85%, and the symptoms of potato tubers were observed 48 h later. Sterile water, elution, and purified protein were boiled for 10 min as control and all processing was repeated 3 times.

###### 2.2.3.5.2 Tuber slices method

Potato tuber slices were tested based on the method of Loria et al. (1995) with some modifications. Healthy potato tubers were selected, washed with tap water, peeled, and soaked in 75% alcohol for 30 s for surface disinfection. Potato tuber slices with a diameter of 1.3 cm and a thickness of 4 mm were made with a sterile hole punch and placed in Petri dishes containing sterile filter paper with 7–8 pieces per dish at 28°C and relative humidity greater than 85%. Sterile water, elution, and purified protein were boiled for 10 min as control and all processing was repeated 3 times.

#### 2.2.4 Construction of *CP966 RS08110* gene mutant and function verification

##### 2.2.4.1 Construction of knockout vector

###### 2.2.4.1.1 Construction of sg-pTRIA plasmid

Spacer sequences containing sticky-end were designed using Benchling,^10^ and the off-target risk of candidate sgRNAs was assessed by CasOT to select sgRNAs with higher specificity. The sequences were synthesized by Biology, and the products of the annealed oligonucleotide strands were obtained by phosphorylation and annealing polymerization reactions. The plasmid pTRIA was digested with Type-IIS restriction endonuclease Bael, dephosphorylated and added into the reaction system with the annealed oligonucleotide duplexes at 3:1 mol, ligated by T4 ligase and then transferred to *E. coli* DH5*α*, which coated on LB containing Apr (50 μg/mL), and incubated at 30°C. Finally, *E. coli* DH5α (sg-pTRIA) was obtained by colony PCR amplification and sequencing.

###### 2.2.4.1.2 Construction of S-D-pTRIA plasmid

*S. galilaeus* 5T-1 genomic DNA was used as template, Up-F/Up-R and Down-F/Down-R were used as primers to amplify the upper arm (Up) and lower arm (Down), respectively. After recovery and purification, donor was obtained by SOE-PCR, which was ligated by T-A cloning into T-loads, and then transformed into *E. coli* DH5 α, which was coated on LB containing Amp (100 μg/mL), and screened for positive transformants by colony PCR using M13-RV/M13-M4, which was named *E. coli* DH5α (donor-T). The extracted plasmids sg-pTRIA and donors-T were digested with Hind *III*, then the purified products were recovered by dephosphorylation with Antarctic Phosphatase, and then ligated by T4 Ligase and transferred into *E. coli* DH5α, which was coated on LB containing Apr (50 μg/mL) and incubated at 30°C for 2 d, finally, the knockout vector *E. coli* DH5α (S-D-pTRIA) was successfully obtained by screening positive transformants by colony PCR and sequencing verification, which transferred to the wild strain *S. galilaeus* 5T-1 by conjugal transfer, and the positive transformants were screened by PCR amplification using the primers aac-F/aac-R for the Apr resistance gene and the primers sg-F/sg-R for the sgRNA framework.

##### 2.2.4.2 Screening of deletion mutant strains

The correctly sequenced transformants were inoculated into TSB medium containing 4 μg/mL thiostrepton and 2 mM theophylline to induce Cas9 expression, then coated to the resistant medium after 3 d of incubation, 24 transformants were randomly taken for initial PCR verification with the external primer F2/R1 of the gene to be knocked, and then further verified with F1R1/F2R2/F3R3 as primers. A mutant strain with successful knockout of *CP966_RS08110* gene was selected and inoculated into non-resistant MS medium, and incubated at 42°C for 2–3 generations to remove plasmid S-D-pTRIA. Single colonies after high temperature incubation were streaked on Apr-containing resistant and non-resistant plates for initial screening, and transformants that grew on the non-resistant plate but not on the resistant plate were selected for PCR validation, in order to screen for deletion mutants without exogenous vectors.

##### 2.2.4.3 Functional verification of *CP966 RS08110* gene

###### 2.2.4.3.1 Phenotypic changes of mutant strain

*(1) Observation of colony phenotype.* Single colonies of wild strain *S. galilaeus* 5T-1 and mutant strain △PL-08110 were inoculated into MS medium, respectively, and incubated in an incubator at a constant temperature of 30°C, protected from light, to observe the morphology of the colonies and pigmentation changes.

*(2) Measurement of growth curve.* Single colonies of wild strain *S. galilaeus* 5T-1 and mutant strain △PL-08110 were inoculated into TSB culture, respectively. After overnight culture, the OD_600_ value of the bacterial solution was adjusted to be consistent, and then inoculated into fresh TSB at 1% inoculum, with three replications, and cultured at 30°C and 180 rpm. The OD_600_ value was measured by ultraviolet spectrophotometer every 2 h.

###### 2.2.4.3.2 Utilization of pectin

10 μL of wild strain *S. galilaeus* 5T-1 and mutant strain △PL-08110 were, respectively, inoculated onto the medium for pectinase activity assay, 2.5 mol/L H_2_SO_4_ was added to the medium after incubated at 30°C for 6 d, immersed for 15 min to observe the formation of hyaline circles and measure their sizes, with three replications.

###### 2.2.4.3.3 Detection for pathogenicity of mutant and wild strains

*(1) Potato tubers method.* Healthy potatoes were selected, washed and soaked in 75% alcohol for 1 min for surface disinfection, sterilized insect needles were used to create micro-wounds, and the micro-wounds were inoculated with 0.5 cm mycelium plug that had been grown on MS medium for 7 d, then placed on petri dishes containing moistened sterile filter paper. Sterile agar plugs were used as a control and repeated three times. They were incubated at 28°C with a relative humidity of more than 85% and to observe the onset of the disease.

*(2) Tuber slices method.* Healthy potatoes were selected, peeled and washed, then surface sterilized by soaking in 75% alcohol for 30 s. Tuber slices with a diameter of 1.3 cm and a thickness of 4 mm were prepared with a sterile perforator and placed in petri dishes containing moistened sterile filter paper, with 6 chips per dish in 3 replications. Inoculation of 0.5 cm mycelium plug that had been grown on MS medium for 7 d in the center of the potato chips was carried out and incubated at 28°C with relative humidity greater than 85%, using sterile agar plugs as a control, to observe the onset of the disease.

*(3) Radish seedlings method.* The strains were inoculated in TSB medium and incubated at 28°C, 180 rpm to prepare a bacterial suspension with a concentration of 10^8^ cfu/mL. The surface of radish seeds was sterilized with 75% alcohol and rinsed with sterile water for 3 times, dried and then placed in Petri dishes containing moist sterile filter paper for germination. 24 h later, the seeds with consistent growth were selected and placed on medium containing water agar, inoculated with 200 uL of the bacterial suspension, and the inoculated TSB medium was used as a blank control, with 3 replications, and the onset of disease was observed after 6 d of culture.

## 3 Results

### 3.1 Bioinformatics analysis

#### 3.1.1 Analyze the physical and chemical properties of 08110 protein

The physicochemical properties of the protein were analyzed by the online ProtParam tool, the results showed that the total length of *CP966_RS08110* gene was 1,248 bp, encoding 415 amino acids, protein molecular formula C_1941_H_2998_N_580_O_612_S_4_, the molecular weight of the expressed protein was 44.38 kDa, theoretical pl was 5.80. In the amino acid composition, the largest percentage was Ala (12.5%), while the smallest was Met (0.2%), and it did not contain Ply and Sec, there are 51 negatively charged residues Asp and Glu and 39 positively charged residues Arg and Lys. The instability index of this protein is 14.50, which is much less than 40, and the isoelectric point is less than 7, so it is assumed that this protein may be an acidic stable protein.

**Figure 1:**
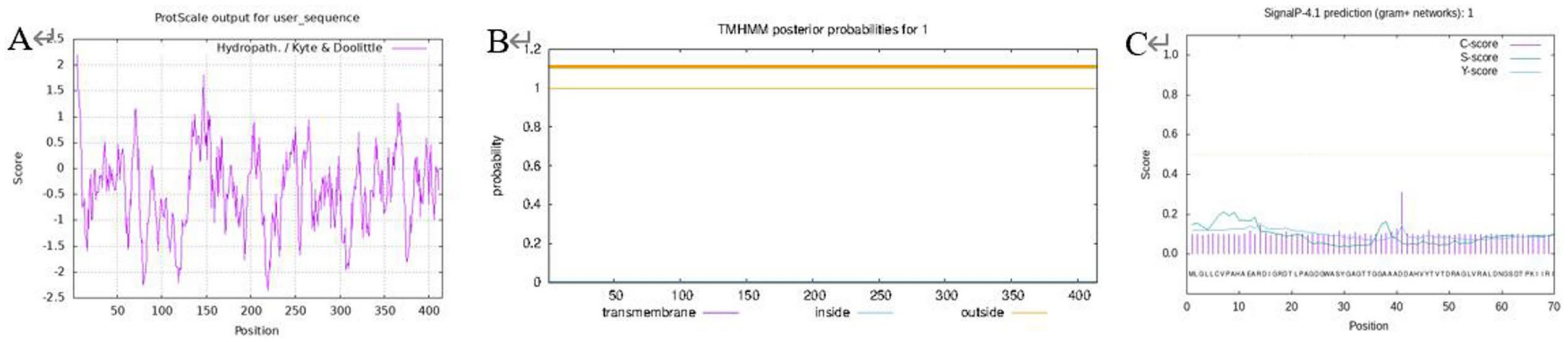
Prediction for protein hydrophilicity/hydrophobicity (A); transmembrane structure (B); protein signal peptide (C).

The hydrophobicity of the protein was analyzed using ProtScale, and the results (Figure 1A) showed that the peak value of Leu at 5th was the highest (2.200), which was the most hydrophobic, and the peak value of Pro at 219th was the lowest (−2.367), which was the most hydrophilic, and the negative regions of the protein were much larger than the positive regions, which indicated that the protein can be considered as a soluble protein.

A prerequisite for the formation of the transmembrane region is that the amino acids in this region have relatively strong hydrophobicity to ensure that the membrane protein can cross the phospholipid bilayer of the membrane. TMHMM was used to predict the transmembrane domain to further verify this conjecture (Figure 1B), the transmembrane signal did not fluctuate and there was no transmembrane helical structure in the encoded product, hence the protein was a non-transmembrane protein. A signal peptide is usually used to guide protein transmembrane transfer or localization, and the prediction of this protein signal peptide using SignalP (Figure 1C) revealed that this protein did not have a signal peptide. It is speculated that it is not a secreted protein or there is no protein transfer involved in the isomerization reaction, so there is no transmembrane structure. This outcome is consistent with the prediction of the transmembrane structure for the proteins mentioned above.

#### 3.1.2 Prediction of protein advanced structure

**Figure 2:**
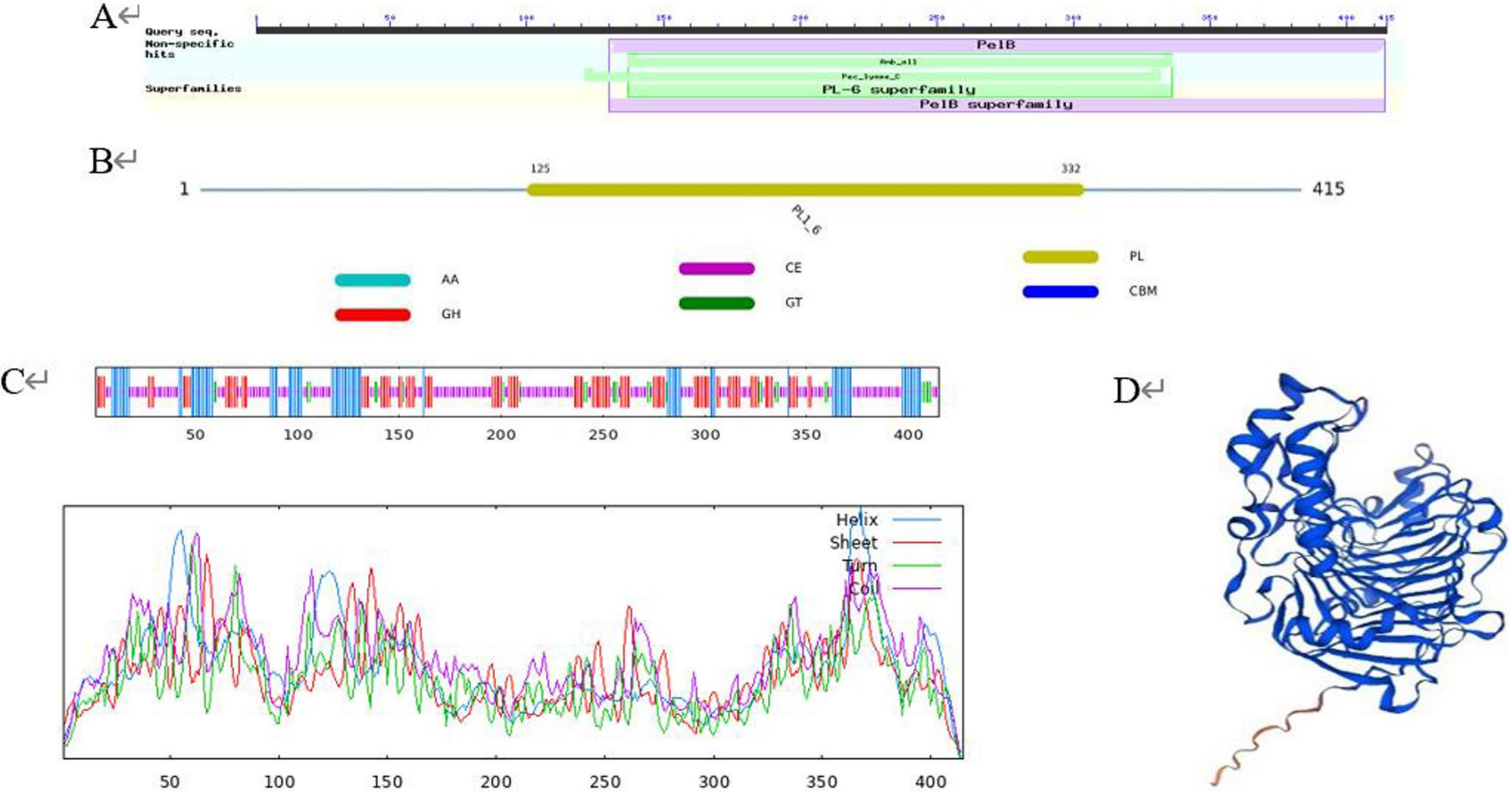
Protein advanced structure. (A) Conserved domain prediction using the CDD database; (B) conserved domain prediction using the dbCAN database; (C) secondary structure; (D) tertiary structure.

SOPMA was used to predict the secondary structure, and the results showed that the protein encoded by PL gene was composed of 19.28% *α*-helix, 25.30% extended chain, 6.75% *β*-fold, and 48.67% random curl, indicating that most of the polypeptide chains of the protein were curled (Figure 2C). Conserved domain prediction using the CDD database showed that the protein contained a complete PL-6 superfamily domain and a Pec_lyase_C domain, forming a right-handed β-helix structure (Figure 2A). PL-6 superfamily is a unique structural domain of the PL1 family. At the same time, the dbCAN database indicated that the protein belonged to the PL1-6 family (Figure 2B). The Swiss model was used to construct the tertiary structure, and the results showed that the protein was constructed using PL A0A0M9YPQ4.1.A of Streptomyces MMG1533 as the template. The sequence consistency between the two was 89.16%, and the GMQE was 0.96. It is proved that A0A0M9YPQ4.1.A is most similar to *CP966_RS08110* in the known crystal structure of PL (Figure 2D).

#### 3.1.3 Phylogenetic tree of protein

**Figure 3:**
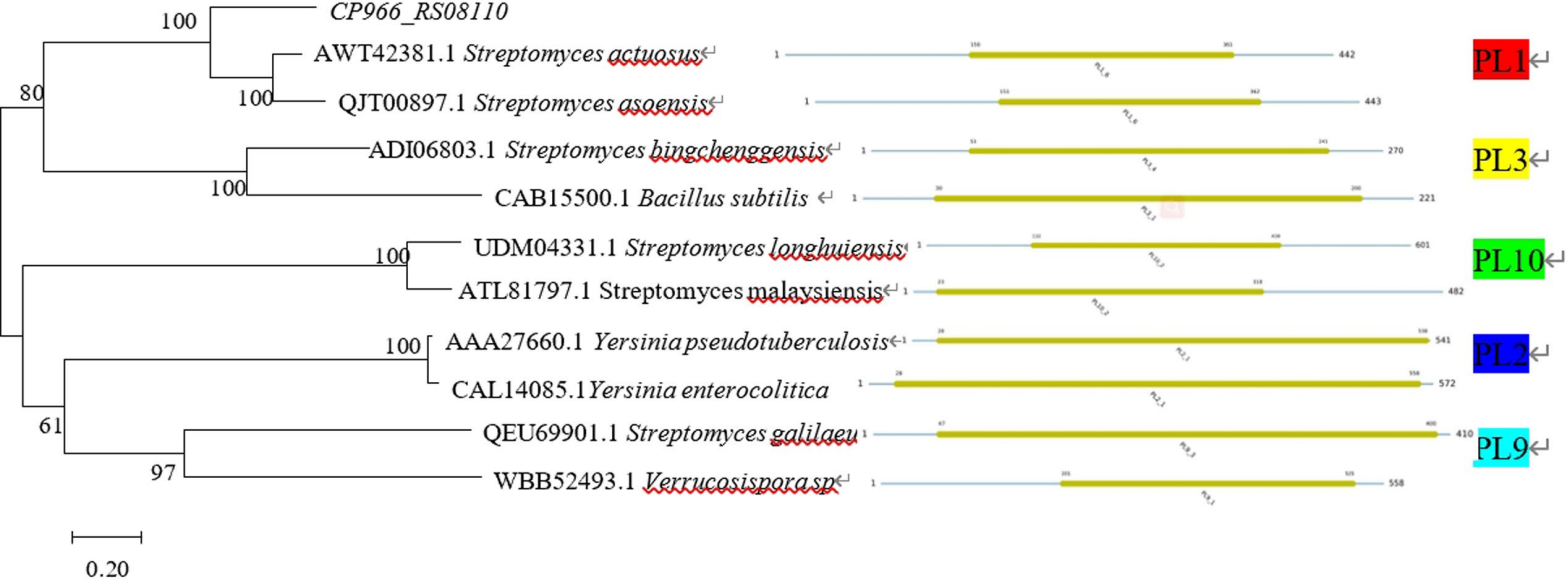
Phylogenetic tree of *CP966_RS08110* gene constructed using the neighbor-joining (NJ) method.

The sequences for the various PL families were downloaded from the CAZY database^11^ and the phylogenetic tree of proteins was constructed using MEGA 7.0. The findings demonstrated that *CP966_RS08110* was a member of the PL1 family since the protein 08110 it encoded belonged to the same evolutionary branch as the PL1 family and had the closest evolutionary relationship (Figure 3). This conclusion is consistent with the results of conserved domain analysis.

### 3.2 Gene cloning

**Figure 4:**
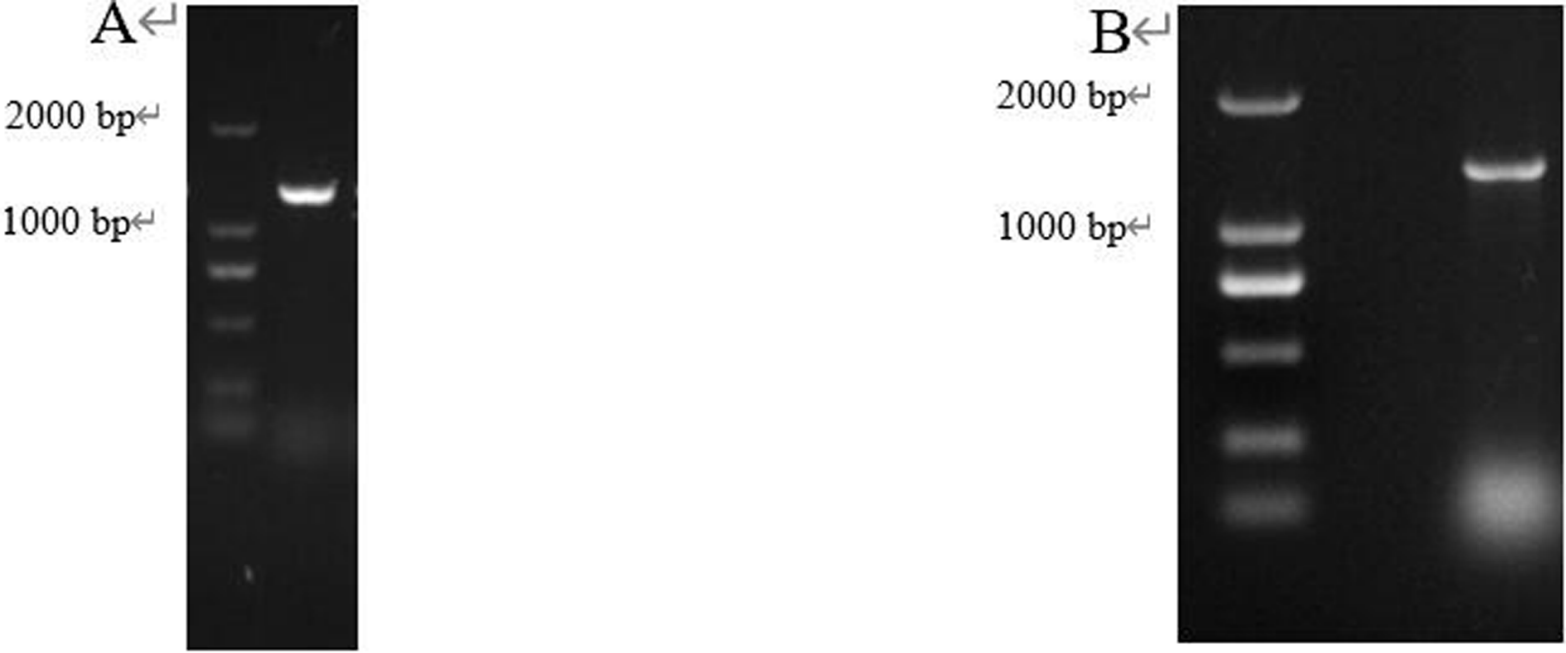
The gene fragment identified using electrophoresis. **(A)** Target gene *CP966_RS08110*; **(B)** Colony PCR.

The target gene *CP966_RS08110* was amplified using the genomic DNA as a template, and a specific band of 1,248 bp was amplified by electrophoresis, which was consistent with the size of the target gene (Figure 4A). The purified target fragment was attached to T-vector, transferred to *E. coli* DH5α, screened by colony PCR (Figure 4B) to determine positive clones, and then sent to Tsingke for sequencing. The sequencing results were consistent with the original sequence, indicating that the gene was successfully cloned.

### 3.3 Recombinant protein expression and pathogenicity determination

#### 3.3.1 Construction of prokaryotic expression system

##### 3.3.1.1 Codon optimization

**Figure 5:**
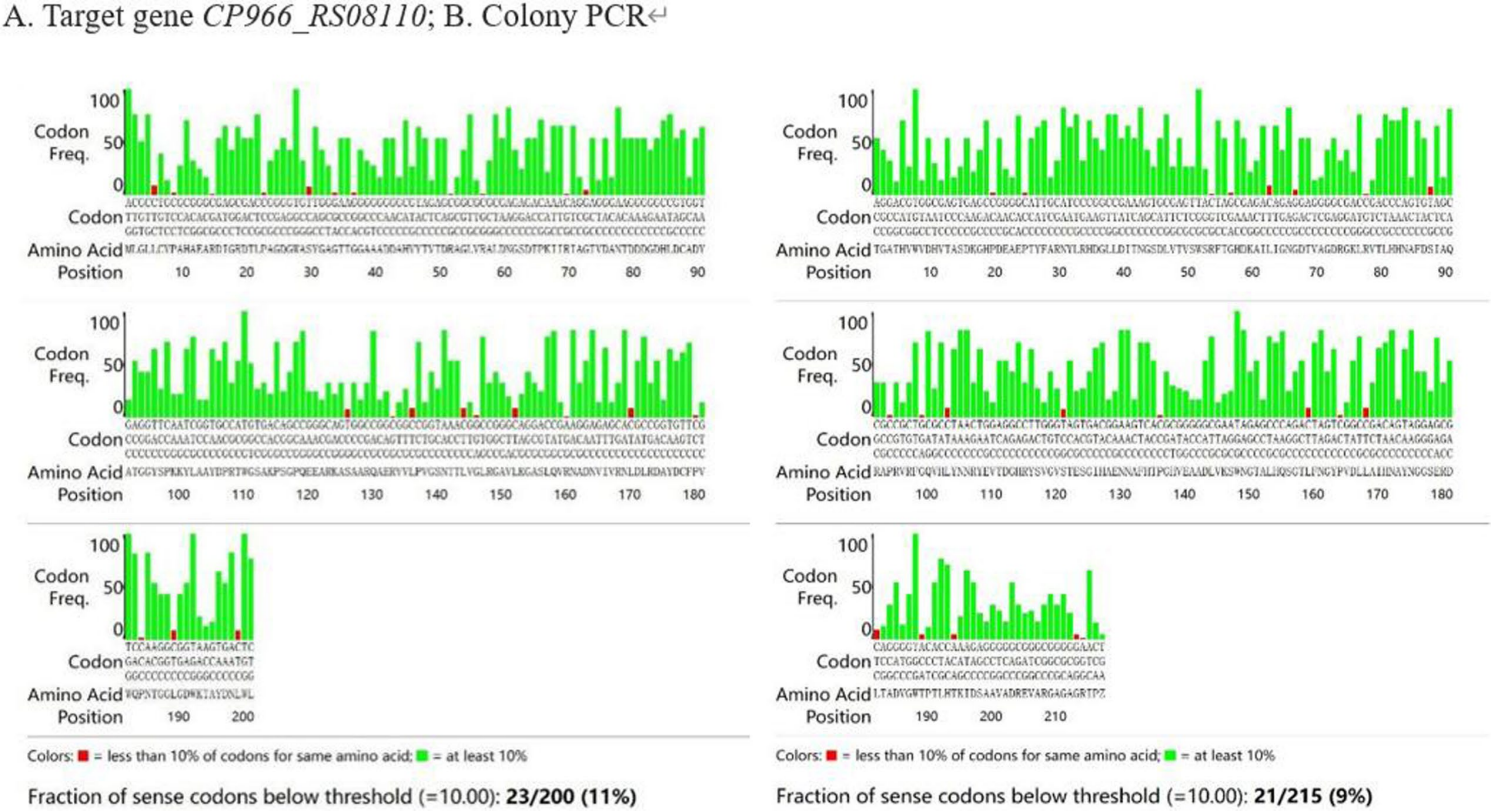
The analysis of codon preference. (A) Target gene *CP966_RS08110*; (B) Colony PCR.

**Figure 6:**
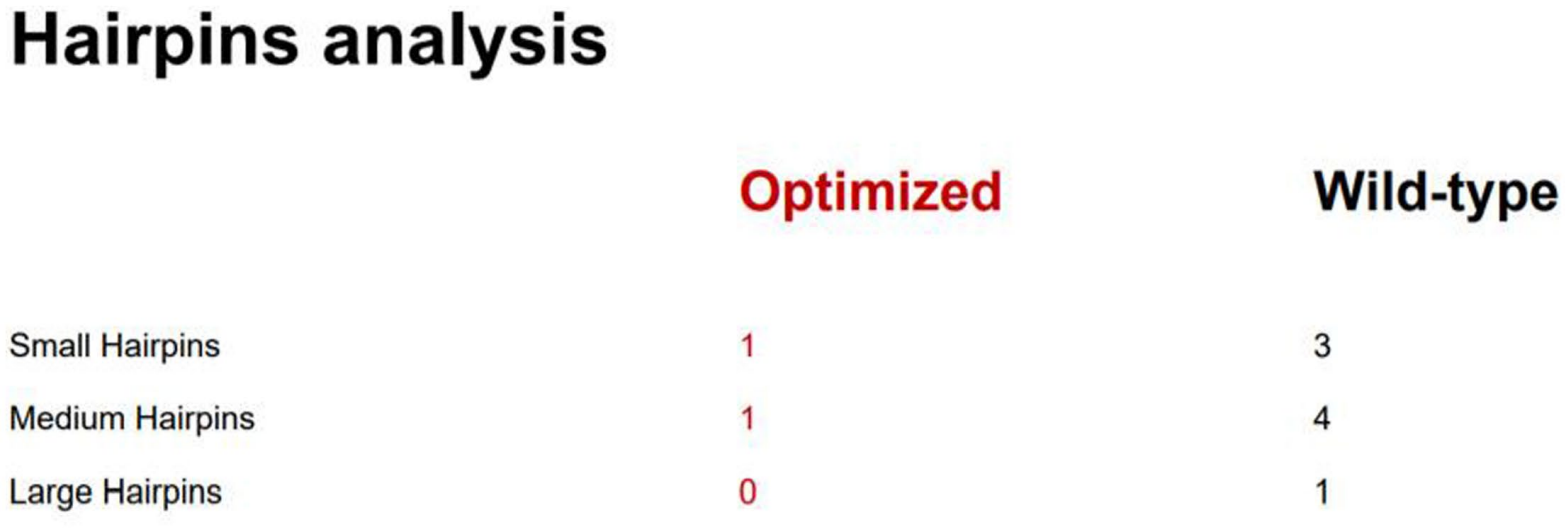
Hairpins analysis.

The frequency of codon usage was analyzed by *E. coli* Codon Usage Analyzer 2.1, the research revealed that 44 codons out of the 415 amino acids were used by *E. coli* for less than 10% (Figure 5). After optimization, CAI increased from 0.36 to 1.00, GC content decreased from 70.59 to 56.33%, and the frequency of codon usage was improved overall. The mRNA secondary structure is another factor that affects the translation process, there were 3 small, 4 medium, and 1 large hairpin structures, respectively, before optimization, after the optimization, there were only 1 small and medium hairpin structure respectively, and 0 large hairpin structure (Figure 6).

##### 3.3.1.2 The prokaryotic expression vector was constructed

**Figure 7:**
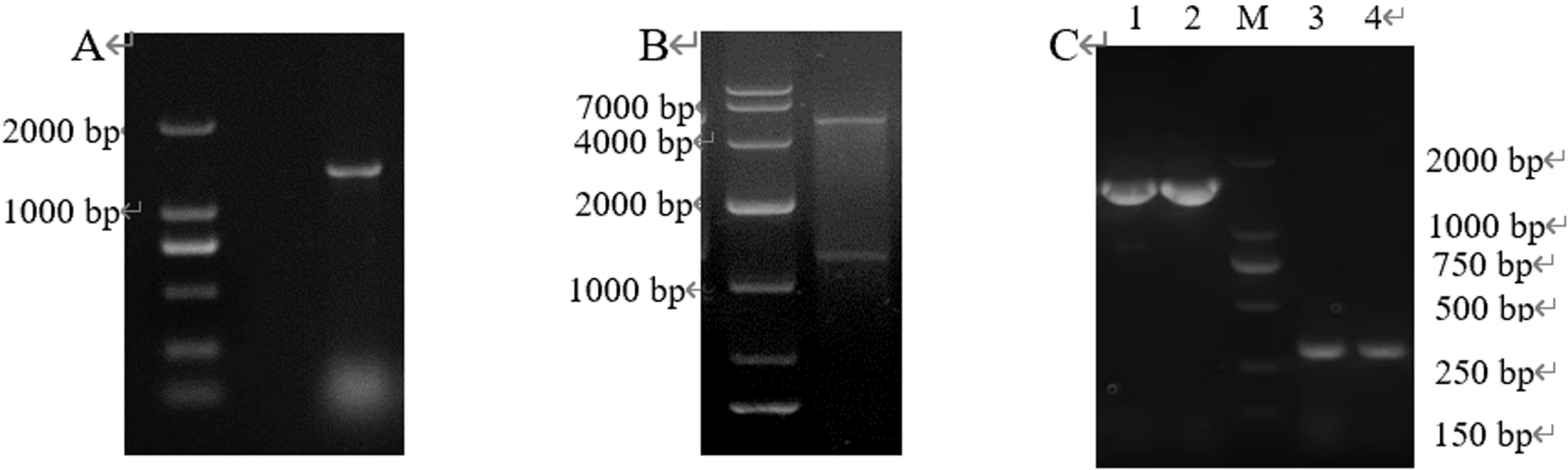
PCR products of positive clones separated by electrophoresis. (A) Colony PCR; (B) Double enzyme digestion; (C) Plasmid PCR (lane 1–2: pET-28a-*New-08110* was used as template and T7/T7 Ter was used as primer; lane 3–4: pET-28a was used as template and T7/T7 Ter was amplified as primer).

Target fragment *New-08110* and pET-28a (+) vector were ligated by T4 ligase, then transformed into *E. coli* BL21 (DE3), which was verified by colony PCR, double enzyme digestion, and plasmid PCR, and the results showed that we have successfully obtained the expression strain *E. coli* BL21 (pET-28-*New-08110*), which can be used for the next experiment (Figure 7).

#### 3.3.2 Induced expression and purification of recombinant protein

##### 3.3.2.1 Growth curve of *E. coli* BL21 (pET-28-*New*-*08110*)

**Figure 8:**
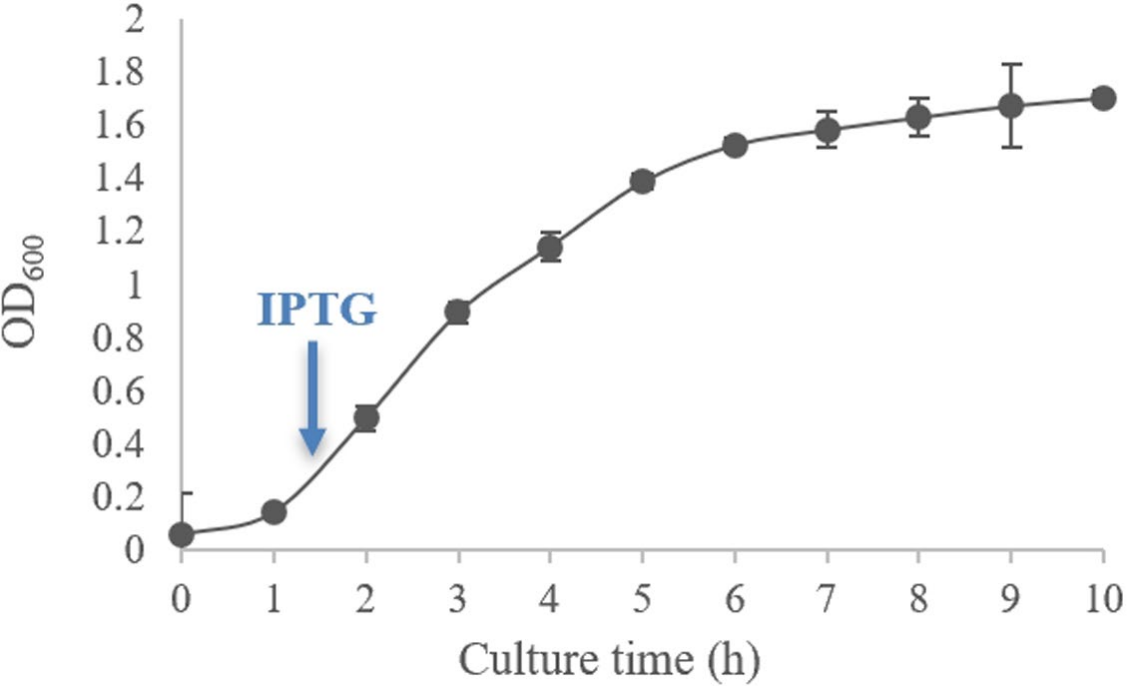
Growth curve of *E. coli* BL21 (pET-28-*New-08110*).

The growth curve of *E. coli* BL21 (pET-28-*New-08110*) is shown in Figure 8. As can be seen from the figure, the logarithmic growth period of the bacteria was between 1–6 h when the bacteria had vigorous metabolism. After culturing for 2.5 h, OD_600_ reached 0.6–0.8, at which time IPTG was added for induction.

##### 3.3.2.2 Determination of recombinant protein

**Figure 9:**
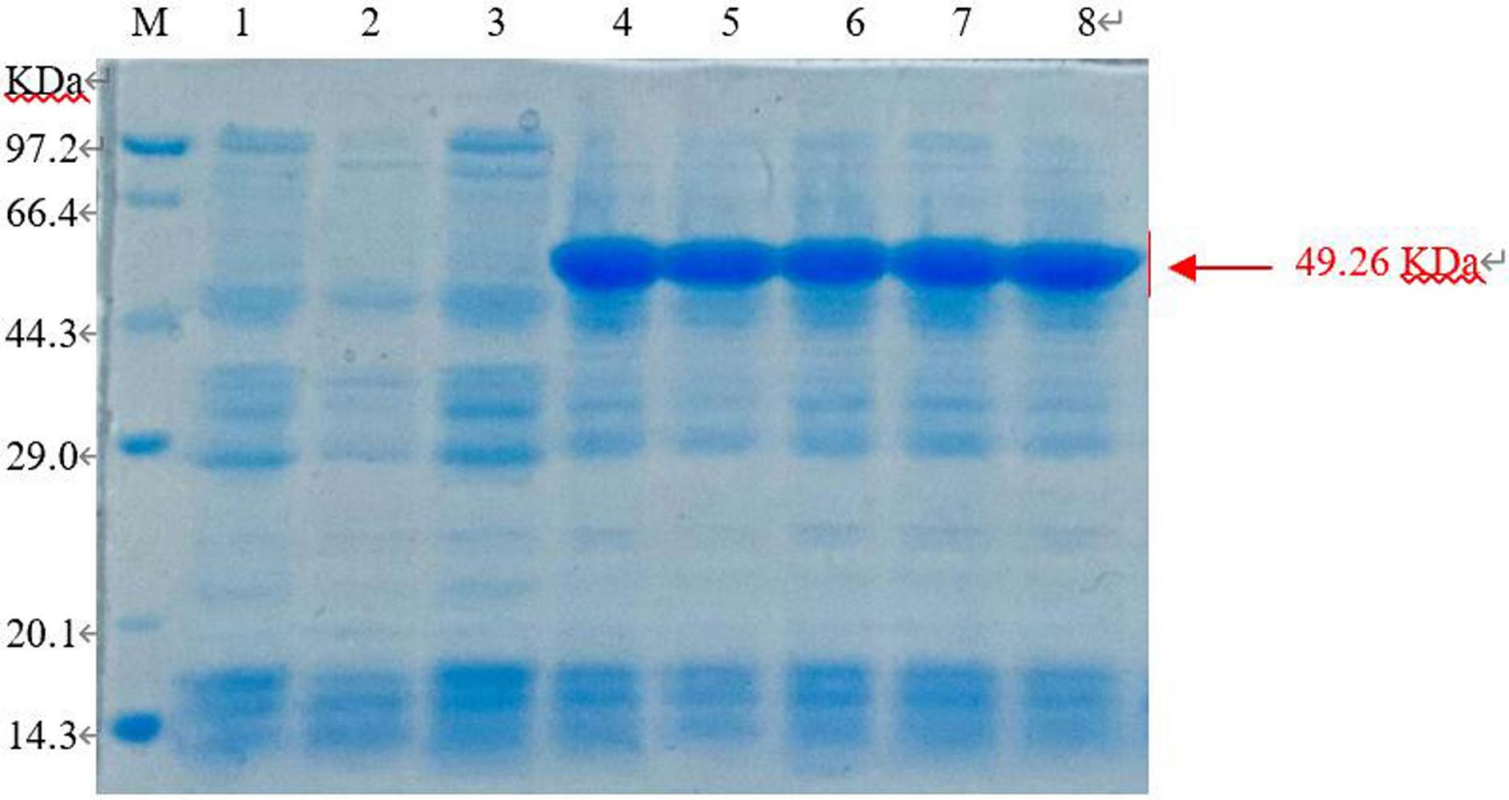
SDS-PAGE analysis of recombinant protein in total sediment (M: Marker; 1. *E. coli* BL21-pet28 not be induced; 2. *E. coli* BL21-pet28 induced at 37°C; 3. *E. coli* BL21 (pET-28-*New-08110*) not be induced; 4–8. *E. coli* BL21 (pET-28-*New-08110*) induced at 37°C).

The *E. coli* BL21 (pET-28-*New-08110*) strain was induced by 0.8 mmol/L IPTG at 37°C for 8 h, and the bacteria were collected. 12% SDS-PAGE electrophoresis showed that specific protein bands were detected at the molecular weight of 49.26 kDa (44.38 + 1.68 kDa) for total protein, whereas no bands were detected in the non-induced strains and no-loaded strains (Figure 9). Therefore, recombinant protein 08110 was successfully induced *in vitro*.

##### 3.3.2.3 Purification of recombinant protein

**Figure 10:**
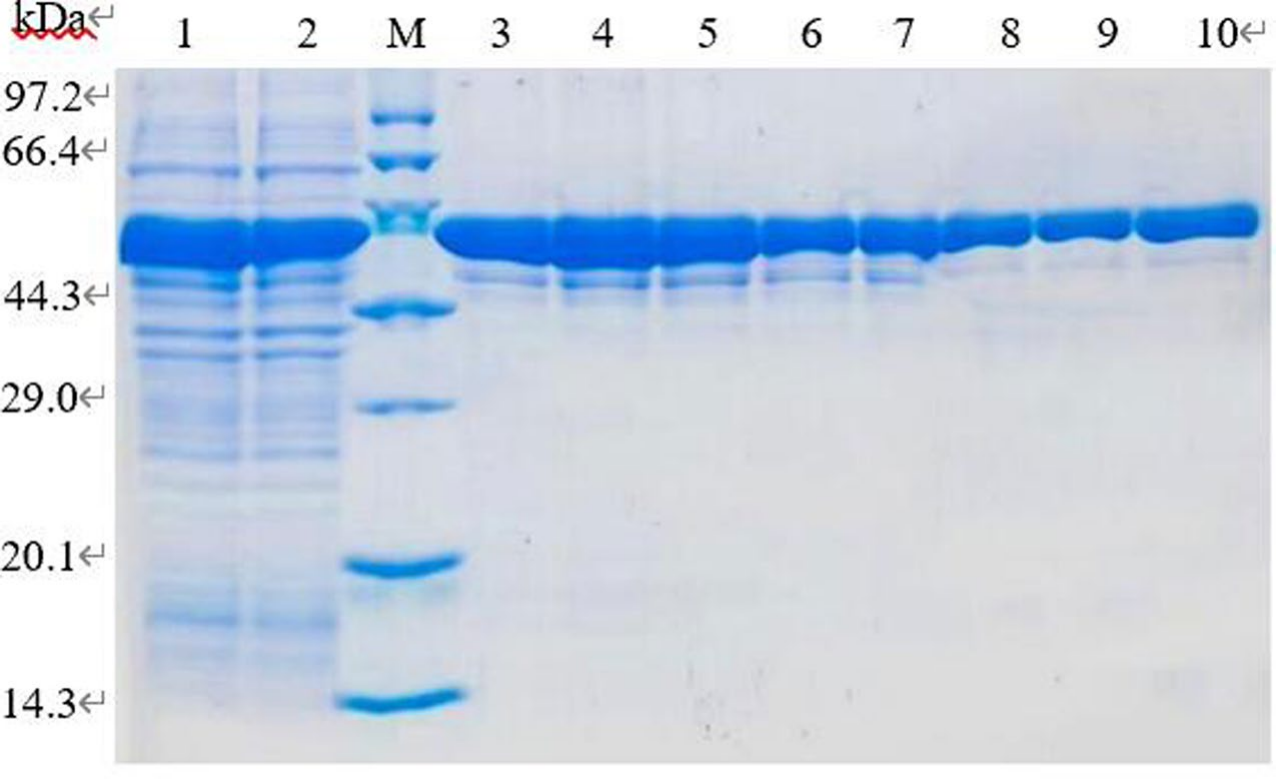
SDS-PAGE analysis of purification of recombinant protein. Lane 1–2. Induced recombinant bacteria lysis supernatant; lane 3–10. Elution of 1–8 times purified protein eluate.

The supernatant induced by IPTG at 16°C and 0.1 mmol/L for 6 h was fractioned by BeyoGold TM His-Purification Resin, the unbound protein was washed away by non-deformable washing solution, and the protein PL was eluted by 500 mmol/L imidazole eluting buffer. The eluted proteins were collected for 12% SDS-PAGE electrophoresis. The imidazole in lanes 6–8 with higher purity was removed by dialysis and subsequent tests were performed (Figure 10).

#### 3.3.3 Pathogenicity test

##### 3.3.3.1 Potato tubers method

**Figure 11:**
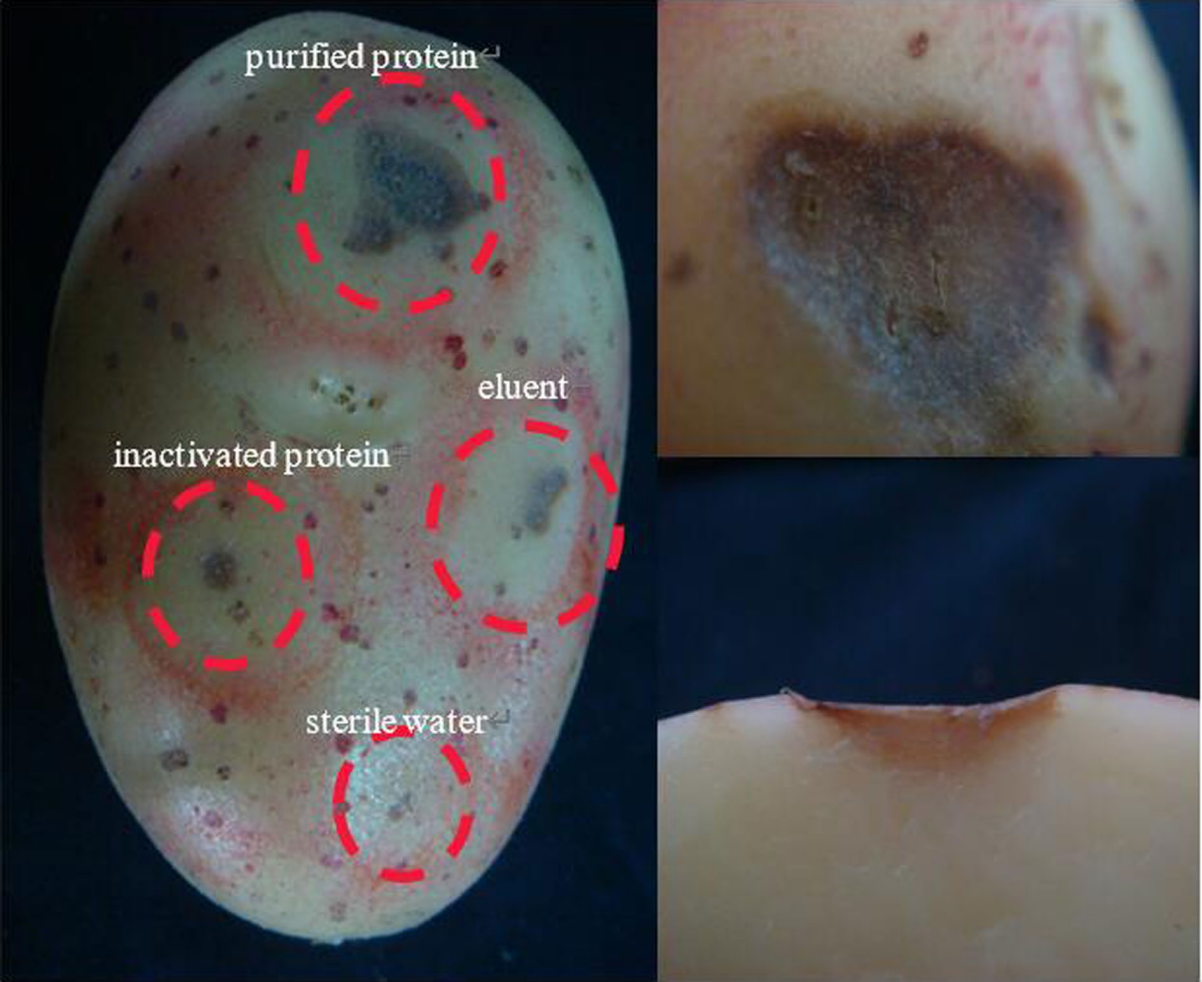
The pathogenicity was determined by potato tubers method.

Recombinant protein 08110 has a strong damaging effect on potato blocks. The potato tubers were inoculated with purified protein by acupuncture. After 48 h, there were obvious necrotic spots, brown, downward concave, obvious edges, and cracks on the spots. None of the controls showed any related symptoms (Figure 11).

##### 3.3.3.2 Tuber slices method

**Figure 12:**
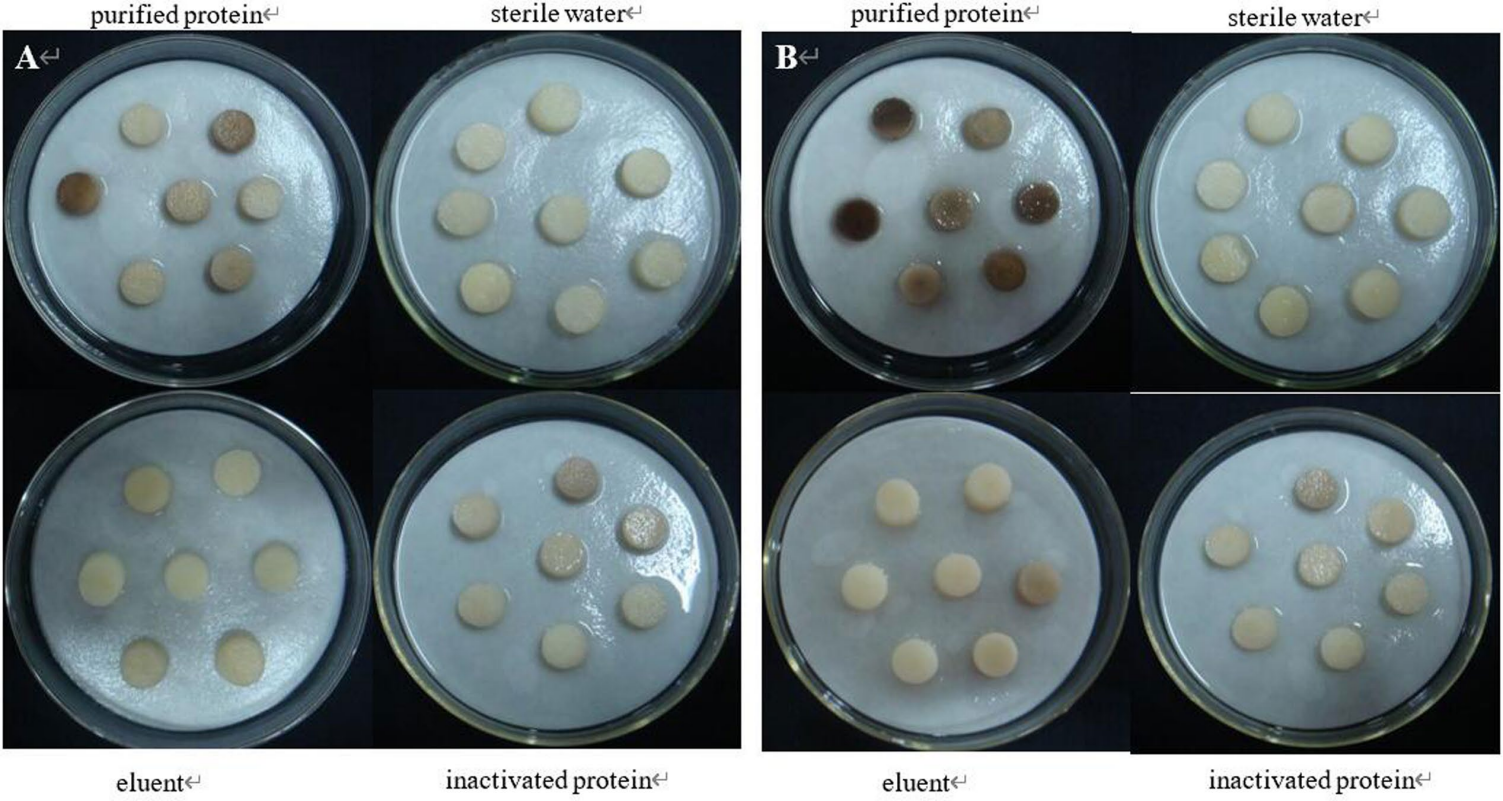
The pathogenicity was determined by tuber slices method. (A) Symptoms at 8 h; (B) Symptoms at 12 h.

The tuber slices were inoculated with purified protein 08110. After 8 h, some tuber slices showed obvious discoloration, but the texture did not change significantly (Figure 12A). After 12 h, tuber slices showed obvious brown lesions, and the texture was softened (Figure 12B).

### 3.4 Construction of *CP966 RS08110* gene mutant and function verification

#### 3.4.1 Construction of knockout vector

**Figure 13:**
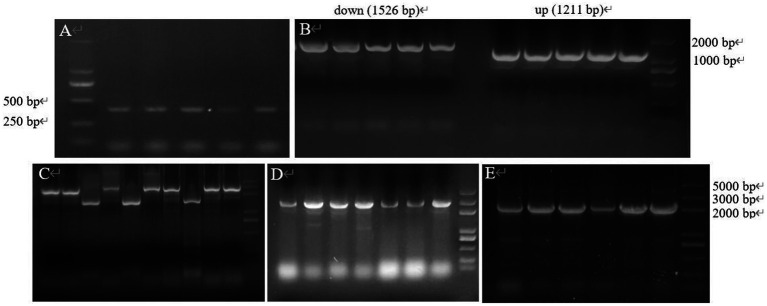
The gene fragment identified using electrophoresis. (A) Electrophoresis of verifying sgRNA; (B) Electrophoresis of up and down arms; (C) Electrophoresis for SOE-PCR of homologous arms; (D) *E. coli* DH5α (Donor-T) colony PCR; (E) *E. coli* DH5α (S-D-pTRIA) colony PCR.

The double-strand oligonucleotide formed by T4 PNK and annealed was ligated into the vector and introduced into *E. coli* DH5α and several transformants were selected from the LB containing Apr-resistant, and colony PCR amplification was performed by using Sg-F/Sg-R (Figure 13A), and the sequence size was obtained by sequencing at 387 bp, which is in agreement with the theory, indicating that the spacer had been successfully inserted into the gRNA scaffold, the sg-pTRIA was successfully constructed.

The specific bands of 1,211 bp, 1,526 bp and 2,737 bp were obtained from the upper and lower homology arms and overlapping PCR amplification, respectively, which were consistent with the theoretical sizes (Figures 13B,C), indicating that the donor was amplified successfully. Donor was ligated with T vector and transferred into *E. coli* DH5α, and colony PCR amplification of the transformants was verified, which resulted in a 2,852 bp band of the same theoretical size (Figure 13D), and the sequencing sequence was consistent with the theoretical sequence homology, which indicated that the recombinant bacterium *E. coli* DH5α (Donor-T) was successfully obtained. The sg-pTRIA vector was ligated to donor by T4 Ligase, and after ligation, it was introduced into *E. coli* DH5α. Colony PCR amplification of the transformants was verified by using primers D-F/D-R, and a single band of about 2000 bp was obtained (Figure 13E), which was consistent with the expected results. Sequencing of the extracted plasmid showed that the sequenced sequence was consistent with the theoretical sequence homology, indicating that the knockdown vector S-D-pTRIA was successfully constructed and could be used for subsequent experiments.

**Figure 14:**
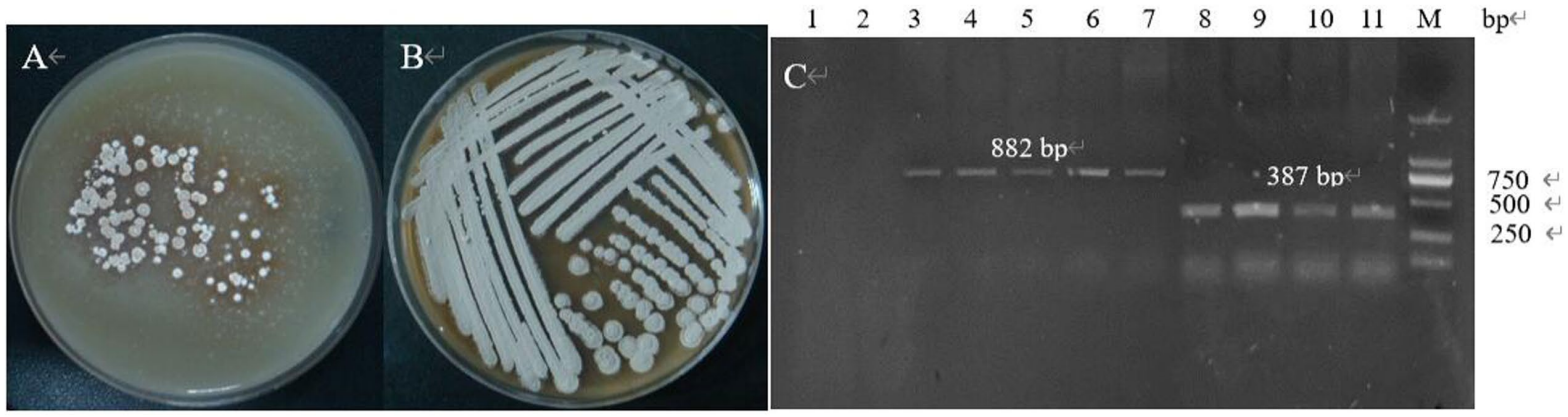
PCR identification of exconjugants. (A) The first generation of exconjugants; (B) The fourth generation of exconjugants; (C) PCR validation (lane 1: acc-F/acc-R was used as primer to amplify the control, lane 2: sg-F/sg-R was used as primer to amplify the control, lane 3–7: acc-F/acc-R was used as primer to amplify the exconjugants, lane 8–11: sg-F/sg-R was used as primer to amplify the exconjugants).

The genetic stability of the plasmid was examined by passaging, and the transformants containing the knockout plasmid S-D-pTRIA (Figure 14A) grew well after three passaging cultures, and no loss of resistance was observed, indicating that the plasmid S-D-pTRIA could be stably inherited in *S. galilaeus* 5T-1 (Figure 14B). The genomic DNA of the transformants was extracted, and PCR amplification was performed using primers acc-F/acc-R and sg-F/sg-R to detect whether the transformants contained the Apr resistance gene and the sgRNA framework, and the results were as shown in Figure 14C. The transformants were all able to amplify an 882 bp fragment of the Apr resistance gene and a 387 bp sgRNA framework, whereas the corresponding size of the wild bacterium was not amplified. Bands, indicating that the knockdown vector S-D-pTRIA had been successfully spliced and transferred to *S. galilaeus* 5T-1.

#### 3.4.2 Screening of deletion mutant strains

**Figure 15:**
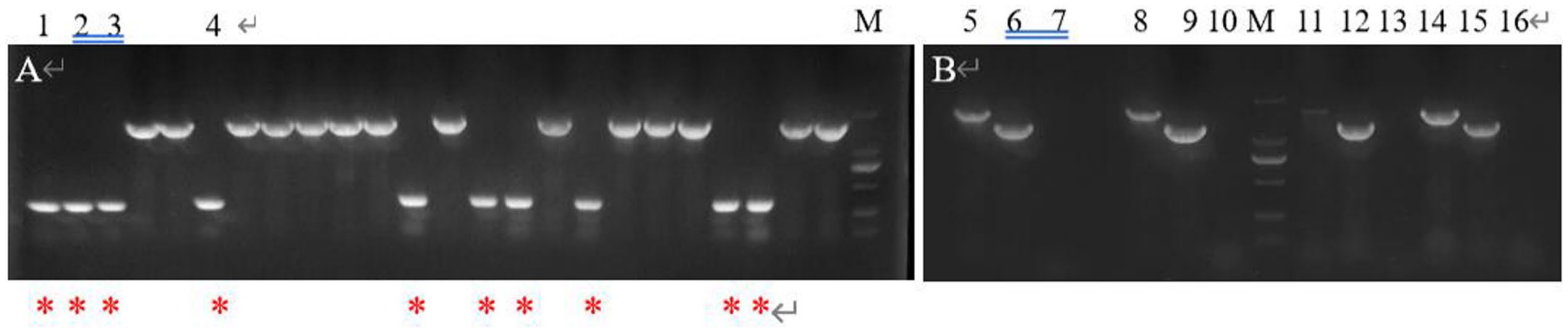
Validation of mutant strain. (A) F2/R1 was used as primer for preliminary validation; (B) 3 primers were used for further verification (lane 5.8.11.14: F1/R1 was used as primer, lane 6.9.12.15: F2/R2 was used as primer, lane 7.10.13.16: F3/R3 was used as primer).

**Figure 16:**
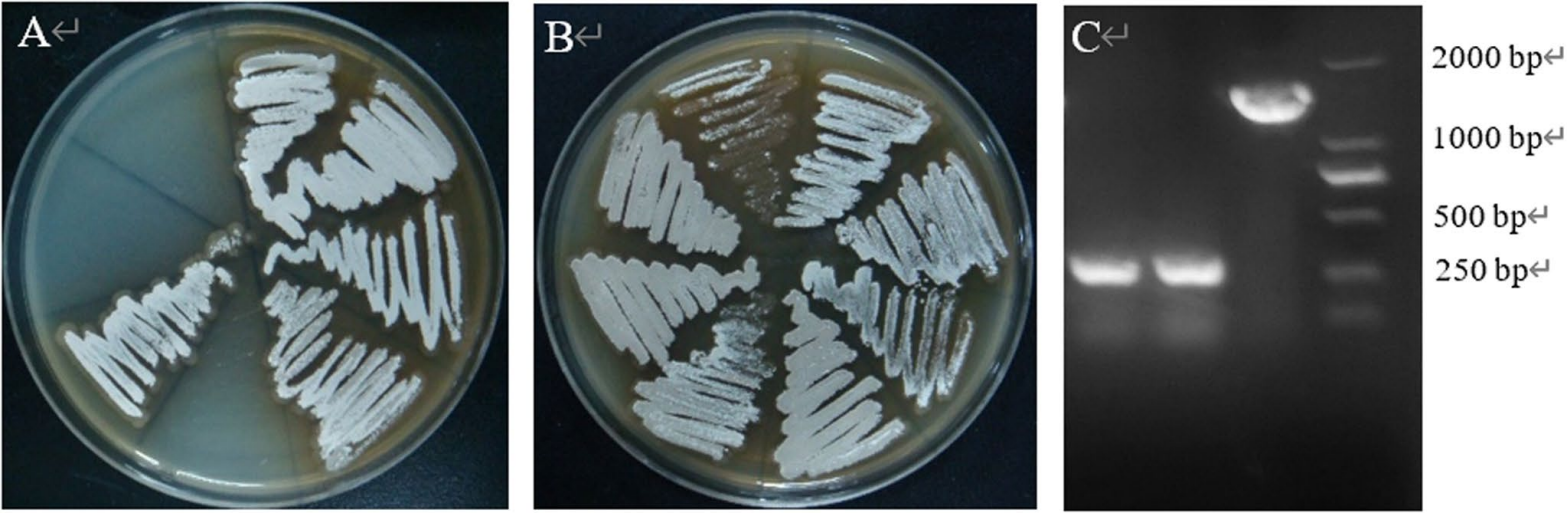
High temperature to remove plasmids. (A) Growth condition on Apr plate; (B) growth condition on non-resistant plates; (C) validation of plasmid loss strains.

After PCR amplification verification, it was found that *CP966_RS08110* gene knockdown was successful in 10 out of 24 transformants (Figure 15A), with a knockdown rate of 41.6%. Mutant strains 1–4 were then selected and further amplified using primers F1/R1, F2/R2 and F3/R3 to verify that F1/R1 amplified a fragment of about 1,500 bp, F2/R2 amplified a fragment of about 1,200 bp, while F3/R3 did not amplify a fragment, which was consistent with expectations (Figure 15B). After high-temperature treatment, it was found that the three transformants did not grow on the resistant plate containing Apr (Figure 16A), while they grew normally on the non-resistant plate (Figure 16B), indicating that the knockout plasmid had been removed. Genomic DNA was extracted and verified by PCR amplification, and one of them recovered the wild-type characteristics during plasmid loss (Figure 16C), finally two plasmid-free *CP966_RS08110* deletion mutant strains were successfully screened, which were designated as △PL-08110.

#### 3.4.3 Functional verification of *CP966 RS08110* gene

##### 3.4.3.1 Phenotypic changes of mutant strain

**Figure 17:**
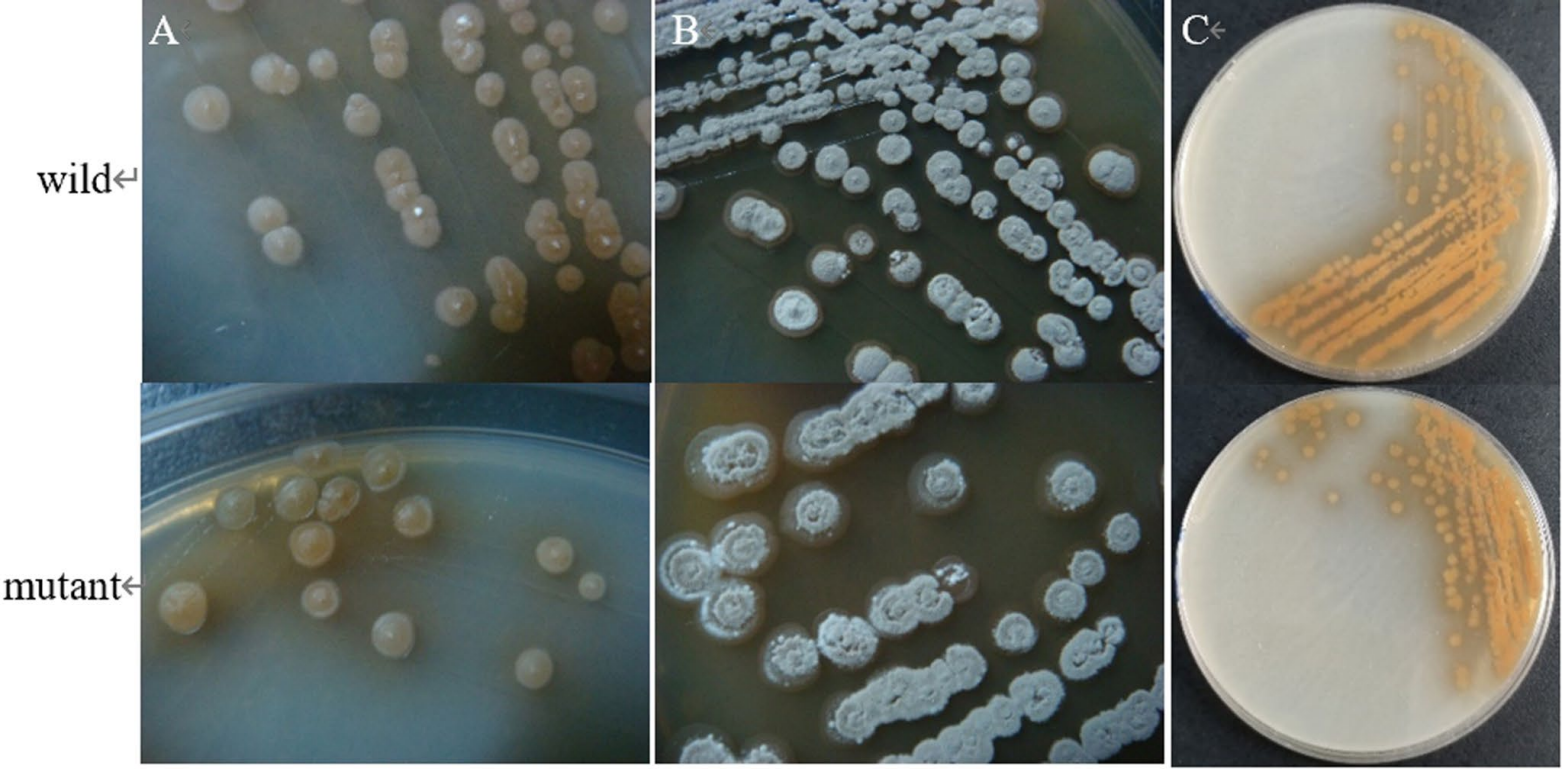
Phenotypic change of two genotype strains. **(A)** The front of the colony at 5 d of culture; **(B)** The front of the colony at 8 d of culture; **(C)** The back of the colony at 5 d of culture.

**Figure 18:**
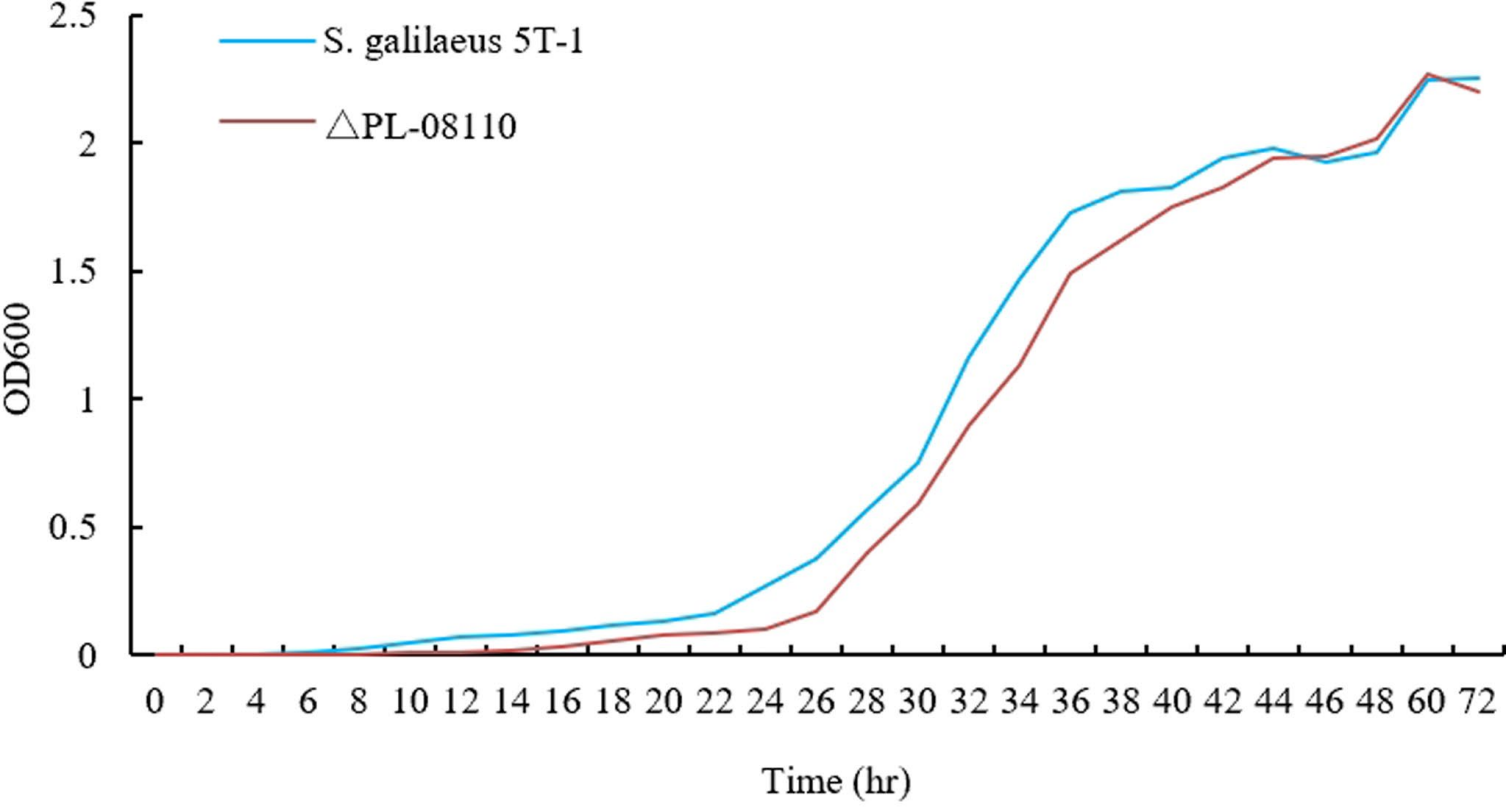
Growth curve determination of two genotype strains.

The results showed that the morphology of the mutant strain and the wild strain on MS medium was consistent (Figures 17A,B) and the pigment production was comparable (Figure 17C), indicating that the knockdown of *CP966_RS08110* had no effect on the morphology of the colonies; the growth curves showed that the wild strain 5T-1 entered into the exponential growth stage at 22 h, and the mutant △PL-08110 entered into the exponential growth stage at 26 h. However, the mutant △PL-08110 had a faster growth rate than the wild strain in the logarithmic growth and stabilization stages, the two strains grew basically the same at 44 h (Figure 18). This result indicated that the deletion of *CP966_RS08110* gene affected the growth rate of the strains in the early stage.

##### 3.4.3.2 Utilization of pectin

**Figure 19:**
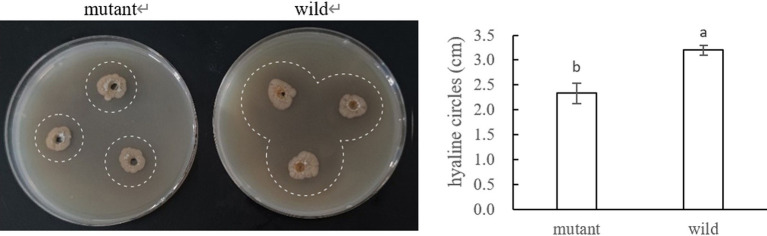
Pectinase activity of the mutant and wide strain.

The results were shown in Figure 19, where the mutant produced significantly smaller hyaline circles utilizing pectin than the wild strain, indicating that the deletion of *CP966_RS08110* led to a decrease in the expression level of the bacterium’s pectinase, which reduced the degradation of pectin-like substances.

##### 3.4.3.3 Detection for pathogenicity of mutant and wild strains

**Figure 20:**
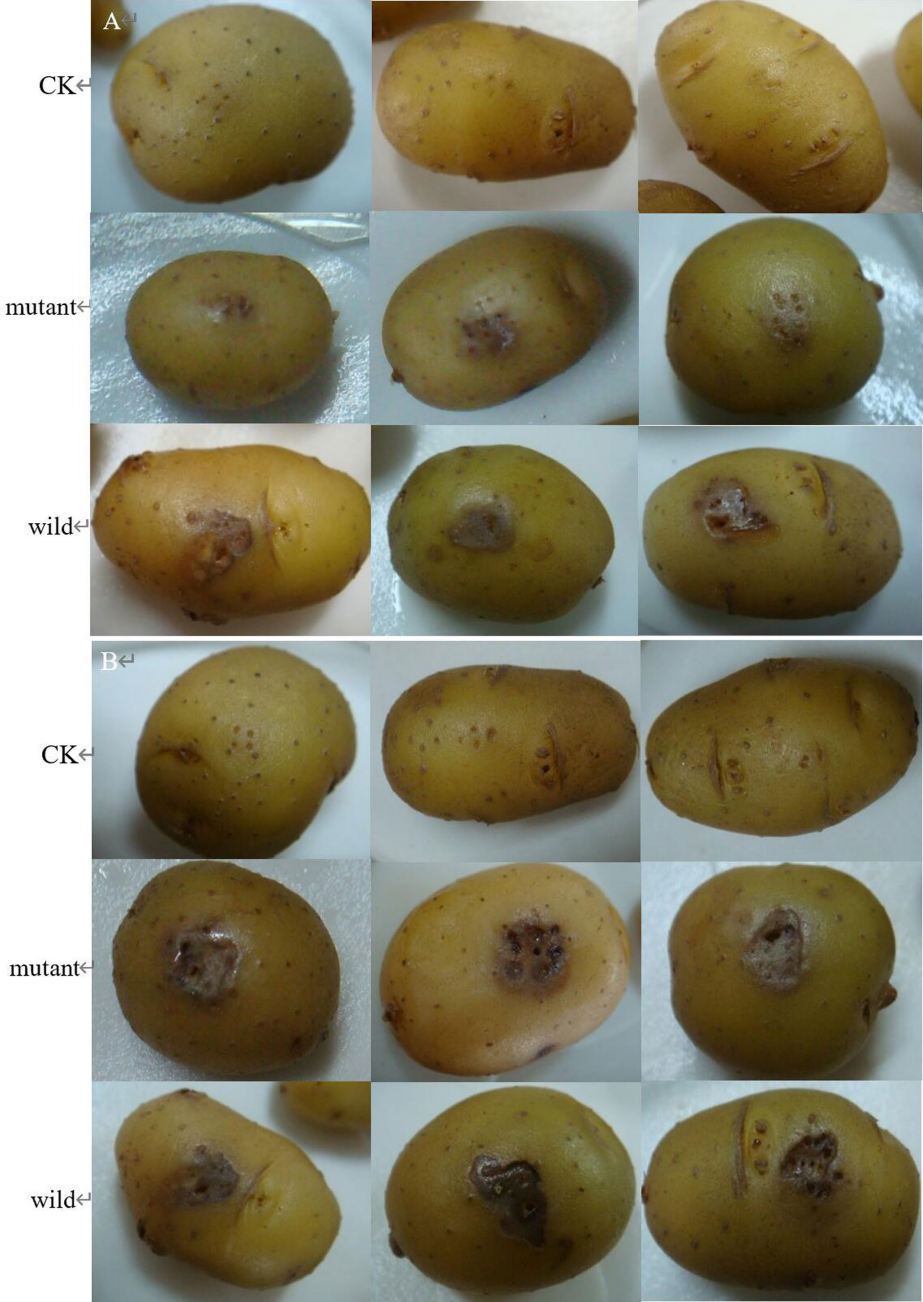
The pathogenicity was determined by potato tubers method. (A) Symptoms at 42 h; (B) symptoms at 5 d.

**Figure 21:**
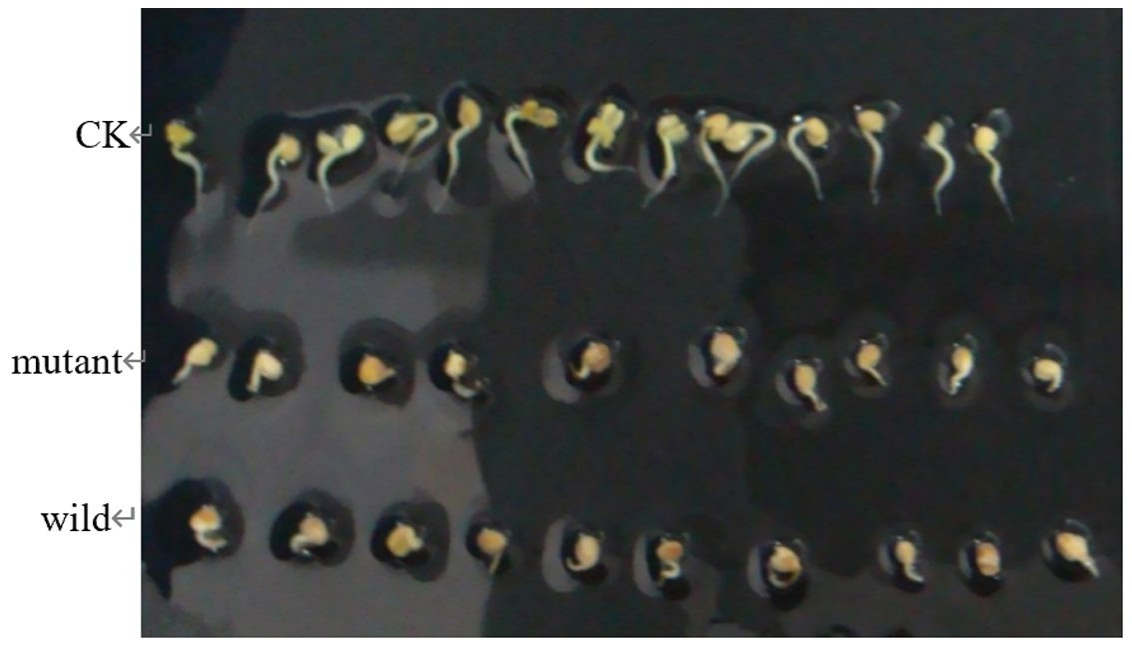
The pathogenicity was determined by tuber slices method.

**Figure 22:**
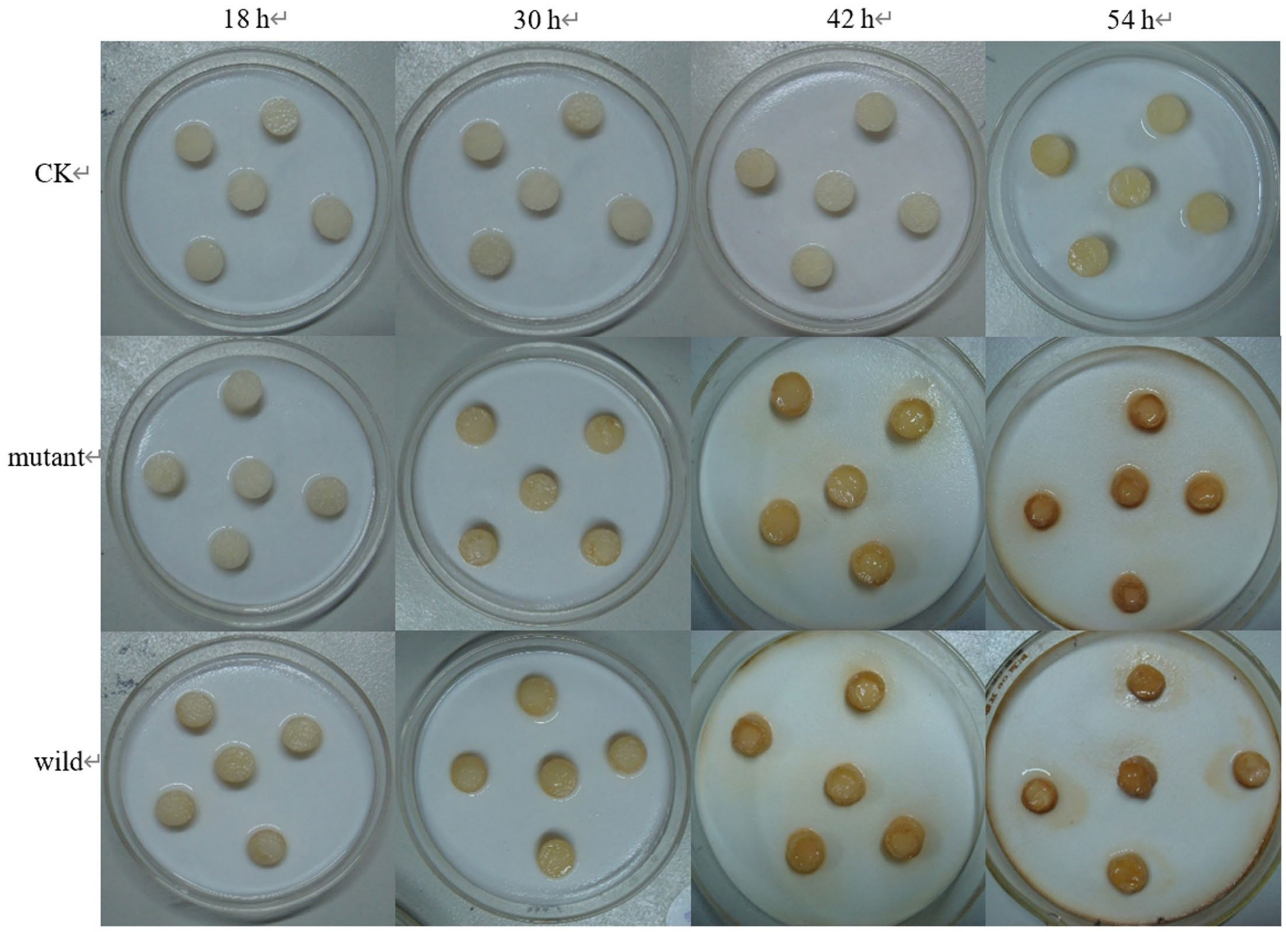
The pathogenicity was determined by radish seedlings method.

The wild and the mutant strain were inoculated into potatoes tubers, and it was found that the inoculation site of the mutant strain began to turn brown at 42 h, while the wild strain had already produced obvious light brown spots at that time; on the 5th day of inoculation, the size of the spots induced by the wild strain and the mutant strain tended to be the same, with browning and necrosis of tissues, and the center of the spot was concave (Figure 20). The wild strain and the mutant strain were inoculated into small potato chips, and it was found that at 18 h, the small potato chips inoculated with the wild strain began to change color, while there was no obvious change between the mutant strain and the control group; at 30 h and 42 h, the chips inoculated with the wild strain and the mutant strain both became light brown, but the color of the lesions induced by the wild strain was darker than that induced by the mutant strain and the softening of tissues was more serious; after 54 h, the two changes tended to be the same, and the small potato chips all became dark brown, with tissue maceration and central depression (Figure 21). The pathogenicity of the wild and mutant strains was determined by the radish seedling method, and the results showed that both the wild and mutant strains could inhibit the growth of radish seedlings, causing symptoms such as stem rot (Figure 22).

In conclusion, the deletion of *CP966_RS08110* affected the infestation rate of *S. galilaeus* 5T-1 but there was no significant difference in the pathogenicity at the later stage, which indicated that this gene was involved in the pathogenicity process of *S. galilaeus* 5T-1, but it might not be the only pathogenic gene of this bacterium.

## 4 Discussion

Numerous studies have demonstrated the significance of PL as an enzyme that breaks down cell walls and is a vital component of numerous pathogenic components of plant diseases. In order to determine whether the *CP966_RS08110* gene is participating in the pathogenic mechanism of *S. galilaeus* 5T-1. In this study, we first analyzed the protein structure encoded by the *CP966_RS08110* gene. The PL-08110 protein, categorized into the PL1 family by a molecular evolutionary tree, includes a unique conserved domain PL-6 superfamily. With a typical parallel *β*-helix, the PL1 family can maintain stability in hostile extracellular settings, and also successfully permeate cell membranes and plant tissues to play a role in glucose metabolism and transport (Zheng et al., 2022). According to reports, Pel C released by *E. chrysanthemi* with the parallel β-helix allowed it to easily pass cell membranes and facilitate its secretion into the extracellular membrane. Furthermore, the topological analysis indicates that Pel C is more likely to penetrate plant tissues due to its special β-helix structure, which enables it to pass through the opening of the dialysis bag even if its molecular weight is theoretically much larger (Herron et al., 2000). Then, we constructed an *E. coli* expression system and explored the most suitable induced expression conditions, and then detected the pathogenic effect of the protein product after obtaining a large amount of soluble protein. The results showed that the *CP966_RS08110* expression product had a significant leaching effect on potato tissues, indicating that this gene may be one of the pathogenic factors of PCS. To further clarify the relationship between this gene and pathogenicity, we subsequently constructed a *CP966_RS08110* gene deletion mutant.

The results showed that the *CP966_RS08110* gene was not related to the morphology and pigment production of *S. galilaeus* 5T-1, but it would affect the pre-growth rate of the strain and the ability to secrete pectinase, and the deletion of this gene affected the infestation rate of *S. galilaeus* 5T-1, but there was no significant difference in the late pathogenicity, suggesting that this gene is involved in the pathogenicity of *S. galilaeus* 5T-1, but it is not the only pectinase-secreting pathogenicity gene of the bacterium, and there may be isoforms. Pectinases usually exist in the form of gene families, and some of the genes have redundant functions (Li W. et al., 2023), and pathogenicity is not dominated by one of the pectinase genes, but rather by the synergistic effect of multiple pectinases, the effect caused by the deletion of individual enzyme genes is eliminated by the substitution of other enzyme genes within the family, and furthermore, there are large variations in the functions of the different members of each of the gene families of the pectinase enzymes, and even if there may be great functional heterogeneity among genes with the same structural domains, and this heterogeneity may lead to pathogenic bacteria showing different pathogenic abilities during host infection (Hong et al., 2019). Although *CP966_RS08110* was knocked out, the activities of other pectinases or cell wall degrading enzymes present in the pathogen were sufficient to maintain its infection, and the deletion of *CP966_RS08110* may also contribute to the increase in the activities of other enzymes, so knocking out one of the pectinase genes did not significantly reduce its pathogenicity.

Fu et al. (2015) reported that there were 12 PL genes in *P. capsici*, but silencing of only 3 genes would directly lead to the decline in the pathogenicity of the pathogen. Tayi et al. (2016) mutated 4 PL genes in *X. oryzae pv. oryzae*, but only the mutant strain with deletion of *PglA* gene completely lost pectinase activity. Dow et al. (1989) pointed out that the absence of PG isoenzyme I in *X. campestris pv. campestris* did not affect the pathogenic ability, and *R. solanacearum* encoded a total of four pectinases, *PehA*, *PehB*, *PehC* and *Pme*, in which the mutation of *PehC* and *Pme* genes did not affect the virulence of the strain (Tans-Kersten et al., 1998; Huang and Allen, 1997, 2000). Those results are basically consistent with our experimental results, indicating that genetic compensation is a universal phenomenon. However, it has also been reported that the deletion of a single PL gene can reduce the pathogenicity of pathogens to their hosts. Yakoby et al. (2001) reported that the pathogenicity of *pelB* mutant of *C. gloeosporioides* decreased by about 40%. Cho et al. (2015) reported that the virulence of *A. brassicicola PL1332* gene deletion mutant was reduced by about 30%. The mutation of *pehA* and *pehB* in *R. solanacearum* strains not only resulted in reduced virulence, but also significantly reduced the frequency and speed of their colonization on tomato stems. In addition, it is worth noting that a completely PG-deficient triple *pehA/B/C* mutant was slightly more virulent than a *pehA/B* mutant (González and Allen, 2003). The reason may be that different pathogenic microorganisms rely on different mechanisms to overcome the host defense system due to differences in pathogenic mechanism and genetic variation. Some pathogens only rely on pectinase to degrade plant cell walls and thus cause diseases, while some pathogens cause diseases through the combined action of more complex and multiple factors. Including exopolysaccharides, toxins, plant growth regulatory substances and effector factors. Of course, in the stage of pathogen attachment and invasion, in addition to its own factors, it also involves the regulation of host and environmental factors. Different plant varieties may have different resistance mechanisms, and environmental conditions such as temperature and humidity will also affect the growth and disease-causing ability of pathogens.

## 5 Conclusion

This work marked the first cloning, expression and acquisition of *S. galilaeus*’s pectate lyase, *CP966_RS08110*, and a deletion mutant strain of this gene has been obtained. Determination of the pathogenicity of the protein and the mutant strain showed that purified protein inoculation of healthy potato tubers can cause brown necrotic lesions, and the deletion of the gene affects the rate of infestation of *S. galilaeus* 5T-1, but there is no significant difference in the late pathogenicity of the ability to indicate that it is only involved in the early stage of the infestation or there is a homozygous gene. The results of this study provide a basis for revealing the pathogenic mechanism of *S. galilaeus*, and lay a foundation for the comprehensive prevention and treatment of scab disease.

## Data Availability

The original contributions presented in the study are included in the article/supplementary material, further inquiries can be directed to the corresponding author.

## Footnotes

^1^https://web.expasy.org/translate/

^2^https://www.expasy.org/resoures/protparam

^3^https://www.expasy.org/resources/protscale

^4^https://services.healthtech.dtu.dk/service.php?SignalP-4.1

^5^https://services.healthtech.dtu.dk/service.php?TMHMM-2.0

^6^https://www.ncbi.nlm.nih.gov/cdd/

^7^https://bcb.unl.edu/dbCAN2/blast.php

^8^https://npsa-prabi.ibcp.fr/cgi-bin/npsa_automat.pl?page=npsa_sopma.html

^9^https://www.expasy.org/resources/swiss-model

^10^www.benchling.com

^11^http://www.cazy.org/
